# The Advantages of Co-Operation in Our Profession

**Published:** 1904-01

**Authors:** Ned A. Stanley

**Affiliations:** New Bedford, Mass.


### THE ADVANTAGES OF CO-OPERATION IN OUR PROFESSION.[1]

BY NED A. STANLEY, D.M.D., NEW BEDFORD, MASS.

Mr. President and Gentlemen,—I have called the subject of this short paper "The Advantages of Co-operation in our Profession." Rather, perhaps, let me say, some of the advantages, for they are many and manifold.

We all know that the profession of dentistry in America is an advanced one. We have for many years, if not from the earliest beginnings, when our profession began to engage the whole attention of able men, led the world in new mechanical devices and methods, which go so far towards the success of a large percentage of the operations we are called upon to perform. To what is our position due? In large part to the co-operation of our practitioners. Everybody is familiar with the power of co-operation as it is felt to-day in the business world. With us the value of co-operation in its best sense is seen; the profession is elevated, made progressive, and every member benefited thereby; the coming together for mutual aid and benefit; the talking over of cases and interchange of ideas; the presentation of some new piece of appliance or method that has simplified difficult operations, and made new ones possible.

In this way the ideas and inventions of individuals have come to be shared by the profession at large. Not only has the operator thus been aided, but his patients have received the direct results of this co-operation.

How much we are indebted to that indefatigable, tireless worker, J. N. Crouse, of Chicago! How many a head that wears a crown rests easier because of the battles he has fought and won for us,—not for *himself*, but for *us!* And how was this made possible? Only by concerted action on the part of dentists in maintaining the Dental Protective Association. The old fable of the bundle of sticks is here well illustrated: "United we stand, divided we fall." And so the profession has grown to be one of the great professions.

---

[1] Read before the Massachusetts Dental Society, Boston, Mass., June 3 and 4, 1903.

Few occupations call for greater skill, a more delicate touch, or better judgment than our own. Our operations as a whole are much more delicate, and call for greater manipulative skill than do those of the surgeon. It is true we do not share the responsibility of the latter, but we are held responsible for our operations and judged accordingly.

I often think what an unsightly lot of humanity we should be, were it not for the dentist. Nothing can destroy the beauty of the face more than an ugly mouth. To be sure we have those distressing, inexcusable exhibitions, where the abuse of the gold cap has seriously marred the features of the patient and erected a monument to the inartistic, fifteen-years-behind-the-times operator.

We, as dentists, have an important function to perform by teaching that part of the public we see professionally—and indirectly many others—how essential is the proper care of the teeth; and particularly so is this true with children. Here is where a very important work should be carried on, and I feel sure it will not be many generations before the inheritance will be a much stronger dental structure. The present condition of our teeth is one of the penalties of civilization. A return to simpler methods of living and moderation in all things will correct many of the unnatural ills from which we are suffering. Now, how can we best carry out this important work intrusted to us? Our patients are entitled to the best we can give them from our own experience and from the experience of others. If we wish to become representative men, we will not feel satisfied with ourselves until we have become acquainted with and mastered the most approved and most artistic methods at our command.

One of the best ways to grasp these rounds of progress is through society meetings, where something new is always to be seen. And the practitioner who is too indifferent—cannot spare the time—or considers that he is self-sufficient is making a mistake. Much can be derived from reading the reports of meetings, but much more can be obtained by being in attendance at the meetings themselves, for things in this world move rapidly. The present German emperor, when still a boy, took for his motto these words: "If I stand still, I rust."

The man who habitually absents himself doubtless spends very little time over dental literature. I would urge the benefits to be derived from these society meetings. To stand still *is* to rust; it is to go backward.

We must keep pace with the progress of the time, or at least keep within sight of the leaders, with always a receptive mind and a pliant hand, to receive and apply new and better methods.

These are some of the advantages to be derived by us from co-operation: Growth, good-fellowship, new ideas, a day off, and you return to your office refreshed, newly aided, and encouraged.

